# Coronary Computed Tomography Angiography to Exclude Acute Coronary Syndrome in Low-Risk Chest Pain Patients

**DOI:** 10.3390/jcdd12120493

**Published:** 2025-12-14

**Authors:** Lauren Ling, Asim Shaikh, Matthew Sibbald

**Affiliations:** 1 Schulich School of Medicine & Dentistry, Western University, London, ON N6A 5C1, Canada; 2 Michael G. DeGroote School of Medicine, McMaster University, Hamilton, ON L8S 4L8, Canada; asim.shaikh@medportal.ca; 3 Division of Cardiology, Hamilton General Hospital, Hamilton Health Sciences, McMaster University, Hamilton, ON L8S 4L8, Canada

**Keywords:** acute chest pain, acute coronary syndrome, coronary computed tomography angiography, emergency department, low-risk chest pain, major adverse cardiovascular events

## Abstract

Background: Coronary computed tomography angiography (CCTA) is a non-invasive imaging tool used predominantly in suspected chronic coronary artery disease (CAD) patients, due to its high negative predictive value. However, increasing focus has been placed on CCTA to manage and risk stratify acute chest pain patients in emergency departments (ED). Objective: This scoping review summarizes the available evidence on the role of CCTA to exclude acute coronary syndrome (ACS) in low-risk acute chest pain patients, focusing on its diagnostic accuracy, safety, and application in the context of high sensitivity cardiac troponin assays (hs-cTn). Methods: Articles published between January 2015 and March 2025 investigating CCTA use in low-risk acute chest pain patients were retrieved from Medline, Embase, Emcare, and Web of Science databases. Results: 22 articles (13,617 patients) were retrieved. CCTA had strong diagnostic performance, with an excellent negative predictive value (99.8–100%) and sensitivity (94–100%) for ACS diagnosis and prediction of major adverse cardiovascular events. Specificity and positive predictive values were lower and less consistent. When combined with hs-cTn, the diagnostic accuracy of CCTA for ACS was improved significantly. CCTA was associated with low rates of ACS at follow-up (0–3.5%), which were lower than or comparable to the safety outcomes of standard care and stress testing.

## 1. Introduction

Chest pain remains a leading cause of emergency department (ED) visits in the United States, with over 7 million cases annually comprising 5.6% of all visits to the ED [1]. Many of these cases are non-cardiac in origin and less than 10% of patients with chest pain are diagnosed with an acute coronary syndrome (ACS) [2,3]. More specifically, despite low-risk chest pain patients (i.e., those presenting with acute chest pain of unknown origin, a non-diagnostic ECG, and negative biomarkers) making up a large proportion of patients presenting to the ED with chest pain, the frequency of ACS or MACE at 30 days in this population remains very low (<1%) [4]. According to recent guidelines on the evaluation and diagnosis of chest pain, routine hospital admissions, stress testing, and invasive imaging are not shown to significantly improve outcomes in this population and instead contribute to substantial resource consumption. These findings underscore low-risk chest pain patients–as opposed to high-risk patients, whose management is already guided by well-established pathways–as a key group in whom rapid rule-out strategies, such as non-invasive CCTA, could improve efficiency and safety outcomes [4].

Traditional diagnostic methods, which typically involve cardiac biomarker testing, electrocardiography (ECG), and clinical assessment, can be lengthy and expensive [5]. Furthermore, they are often limited in their ability to accurately detect or exclude an acute coronary syndrome (ACS) in low-risk patients [6]. Thus, with the lack of diagnostic accuracy offered by current tests, physicians are faced with the challenge of distinguishing between patients who require urgent care and those with less threatening conditions who can be appropriately discharged. Hospital admission and downstream testing expose patients to invasive procedures and has long-term cost and efficiency implications. Conversely, an inappropriate discharge from the emergency department is associated with increased mortality and worsened health outcomes [6,7].

Coronary computed tomography angiography (CCTA), a non-invasive imaging modality, holds promise for the evaluation of chest pain patients, particularly those at low risk for ACS. CCTA provides a rapid assessment of coronary vessel anatomy and plaque burden, which allows for the detection of significant coronary artery disease (CAD) as well as obstructive disease or high-risk plaque features consistent with ACS [8]. Due to its non-invasive nature, CCTA shows potential as an alternative diagnostic tool to invasive coronary angiography (ICA) [9,10].

This scoping review aims to summarize the literature on the use of CCTA in patients with low-risk acute chest pain in the ED setting within the past ten years. The objectives of this study are to examine: (1) the diagnostic accuracy of CCTA compared to other diagnostic modalities; (2) the impact of CCTA on long-term safety outcomes; and (3) the role of CCTA in the context of high-sensitivity cardiac troponin (hs-cTn) assays.

## 2. Methods

We used scoping review methodology to explore the extent of the literature within the past ten years surrounding the use of CCTA to exclude ACS in low-risk acute chest pain patients presenting to the ED. This allowed us to comprehensively summarize the breadth of evidence available and identify significant gaps, without the constraints of conventional systematic reviews [11]. This study was conducted in accordance with the PRISMA extension for Scoping Reviews (PRISMA-ScR) guidelines [12], and is, to our knowledge, the first to synthesize the more recent (past 10 years) body of literature on this topic. The protocol for this review is not registered in PROSPERO or any other protocol database.

### 2.1. Search Strategy

Medline, Embase, Emcare, and Web of Science databases were searched for articles published between January 2015 and March 2025 using the following keywords: computed tomography angiography, acute coronary syndrome, emergency department, and low-risk chest pain. Editorials, case reports, case series, conference abstracts, manuals, letters, books, commentaries, opinion papers, non-human studies, studies not published in English, and duplicates were excluded. The most recent search was conducted on 19 March 2025.

### 2.2. Screening

Articles were independently screened by title and abstract by two reviewers (A.S., L.L.) using Covidence (www.covidence.org (accessed on 19 March 2025)). All reviewers trained on a common set of 40 articles to ensure inclusion and exclusion criteria consistency. Empirical data (cross-sectional, cohort, controlled trials, randomized data, systematic reviews, and meta-analyses) pertaining to CCTA and its use in the emergency department, impact on long-term safety outcomes, and diagnostic accuracy outcomes were included. Studies on the triple rule-out protocol were omitted unless specific data on the exclusion of ACS was collected. Additionally, papers focusing exclusively on efficiency outcomes such as cost-effectiveness, length of stay, and resource utilization, were excluded to maintain the safety outcome focus of this paper. Editorials, case reports, case series, conference abstracts, manuals, letters, books, commentaries, and opinion papers were excluded.

### 2.3. Eligibility and Data Extraction

During full-text screening, each article was independently screened by two reviewers (A.S., L.L.); disagreements were resolved through consensus or a third party (M.S.). Reviews were screened for relevant primary articles published within the last ten years (January 2015 to January 2025). Only studies focusing on low-risk acute chest pain patients (“low-risk” defined as having non-diagnostic ECG and negative conventional troponins) were included for final inclusion in this review. Key characteristics of each study were extracted into a standardized form; primary and secondary outcome results aggregated, analyzed, and presented into three tables by a single reviewer (L.L.).

### 2.4. Ethics Approval

Ethics approval was not required for this study as the data collected was retrieved from previously published studies.

## 3. Results

**Figure 1:**
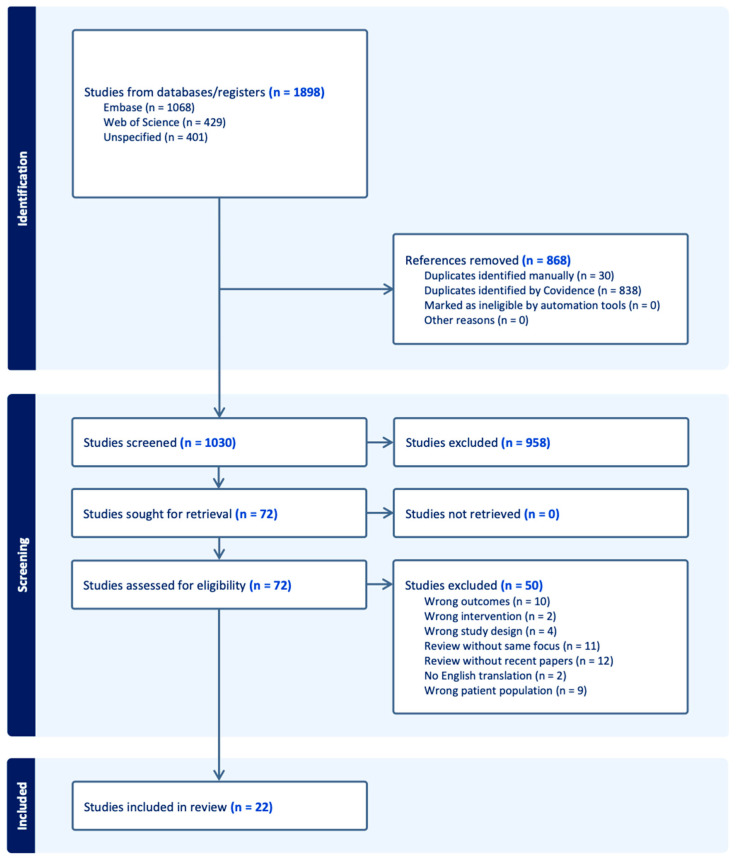
Flow diagram of the scoping review methodology.

The results of the literature search are presented in Figure 1. A total of 1030 articles were identified, 72 articles were included in the full-text review, and 22 met criteria for inclusion [13,14,15,16,17,18,19,20,21,22,23,24,25,26,27,28,29,30,31,32,33,34]. Across these studies, a total of 13,617 patients were included. Key study characteristics, including study design, population size, and outcomes, can be found in Table 1. Detailed CCTA protocols used by each study can be found in Supplementary Table S1.

The patient populations from the retrieved studies included individuals presenting with low-risk chest pain to ED. The age range of patients varied, with most studies including adults aged 18 years and older. The most prevalent risk factors and comorbidities identified were hypertension, dyslipidemia, diabetes mellitus, family history of CAD, and smoking. Elevated biomarkers, ischemic ECG changes, known/previous CAD, renal insufficiency, atrial fibrillation, prior coronary artery bypass grafting or stenting, and contrast allergies were deemed exclusion criteria for most studies. Patient demographic results can be found in Supplementary Tables S2 and S3.

### 3.1. Diagnostic Accuracy

#### 3.1.1. Detecting ACS

Across all studies, the negative predictive value (NPV) of CCTA in detecting ACS was excellent, ranging from 99.8% to 100%, irrespective of stenosis cut-off value (i.e., ≥50% vs. ≥70%) (Table 2). Positive predictive values (PPVs) were far more variable and modest (18–74%), highlighting the low prevalence of ACS among this low-risk population. CCTA had a high sensitivity, ranging from 94% to 100%; specificity values were often lower and less consistent (53–88%). At a ≥50% stenosis cut-off value, both the PPV and specificity were reduced, while NPV and sensitivity increased or remained constant.

A prospective study conducted by Mas-Stachurska et al. compared the diagnostic accuracy of CCTA to stress echocardiography [22]. At a ≥50% stenosis cut-off value, the sensitivity of CCTA was higher than that of stress echocardiography (100% vs. 82.3%, *p* > 0.05), but specificity was lower (76.9% vs. 88.4%, *p* > 0.05)). However, when raised to ≥70%, CCTA performed better than exercise echocardiography across all diagnostic accuracy measures.

Bamberg et al. reported on age- and sex-based differences in CCTA diagnostic accuracy in the detection of ACS, pooling evidence from two randomized controlled trials [27]. In both men and women, NPV and sensitivity remained consistently high across all age groups. Conversely, specificity and PPV decreased with age, and PPVs were notably low (18–35.2%). No significant differences were observed between the diagnostic accuracy of CCTA in men versus women.

#### 3.1.2. Predicting MACE

Three studies evaluated the diagnostic accuracy for the prediction of MACE. These studies used different methods to verify MACE outcomes, including conducting follow-up telephone interviews and comparing it to invasive coronary angiography as the gold standard. The overall diagnostic accuracy of CCTA for the prediction of MACE was high at 91% (Table 2) [24]. NPV was 100%, with only the use of a ≥70% stenosis cut-off yielding a slightly lower value (83%). Galea et al. reported an NPV of 100% in the absence of any obstructive CAD on CCTA [21]. Sensitivity was consistently high (85–100%), while specificity ranged lower (62–92%). Using a ≥50% stenosis cut-off value, PPV was variable, ranging from 33% to 96%.

Only one comparative study was conducted on the diagnostic accuracy of CCTA in predicting MACE. This randomized observational study, by Nabi et al., found that stress myocardial perfusion tomography (SPECT) with stress-only imaging outperformed 50% stenosis cut-off CCTA in terms of both overall diagnostic accuracy (98% vs. 91%, *p* < 0.0001) and specificity (99% vs. 92%, *p* < 0.0001) [24].

### 3.2. Safety Outcomes

#### 3.2.1. ACS at Follow-Up

Thirteen studies reported on the rates of ACS during the follow-up period, which ranged from 30 days to 3 years (median 6 months). In studies where ACS was defined as the occurrence of MI alone, patients who underwent CCTA for acute chest pain experienced a very low rate of ACS across all follow-up durations, ranging from 0% to 3.5% (Table 3). When ACS was measured as a composite of both MI and UA, the event rate was slightly higher (0.5–7.8%).

When measured against standard of care (SOC) and stress testing, CCTA had a lower or comparable event rate. Conversely, compared to stress echocardiography, CCTA was associated with more frequent ACS during follow-up, although the statistical significance of these differences was not mentioned.

#### 3.2.2. MACE at Follow-Up

The rate of MACE was measured in seventeen studies. The definition of MACE was heterogeneous across studies but was often a composite of two or more of the following: cardiac death, ACS, revascularization, and rehospitalization for cardiovascular causes. The criteria used by each study is reported in Table 4. Follow-up duration ranged from 28 days to 4.7 years, with a median follow-up of 6 months.

Overall, rates of MACE were variable, likely attributed to the inconsistent inclusion/exclusion of revascularization and/or rehospitalization as part of the MACE definition. Across all studies, the rate of hard events such as cardiac death, all-cause mortality, and ACS was low, with revascularization being the primary contributor to high MACE rates.

### 3.3. CCTA & High-Sensitivity Cardiac Troponins

When a CCTA-guided strategy was compared directly to standard care encompassing hs-cTn, there was no difference in the rate of MACE (10% vs. 9%, *p* = 0.54), revascularization (9% vs. 7%, *p* = 0.40) or incidence of undetected ACS (0.5% vs. 1%, *p* = 0.54) within the 30-day follow-up period [28].

A nested cohort study in patients enrolled in the ROMICAT II trial assessed the effectiveness of a combined strategy involving hs-cTnI followed by CCTA [20]. The combined strategy significantly increased the diagnostic accuracy for ACS as compared to conventional troponins and CCTA. Sensitivity and NPV remained consistent at 100%; specificity and PPV increased from 48.2% to 68.1%, and 20.7% to 29.7%, respectively.

In low-risk acute chest pain patients with inconclusively elevated high-sensitivity troponins (defined as having hs-cTn concentrations between rule-in and rule-out thresholds), CCTA safely ruled out type 1 NSTE-ACS [18]. Sensitivity, NPV, specificity, PPV were 95%, 98%, 56%, and 35%, respectively, when a ≥50% stenosis cut-off value was used. When only coronary segments with a diameter of ≥2 mm were considered, sensitivity and NPV increased to 100%. CCTA was also able to identify other clinically relevant cardiac and non-cardiac conditions in this population, such as pulmonary embolism, pneumonia, and aortic dissection [18].

The number of major adverse cardiovascular events at 12 months did not differ when SOC versus a CCTA-guided strategy was used in this patient population (6.5% vs. 5.6%, *p* = 0.78). However, CCTA was associated with a decreased rate of outpatient referrals/investigations (*p* = 0.01) and a significant increase in the rate of aspirin prescriptions at discharge (*p* = 0.008) [26].

### 3.4. CCTA Technology

No differences were found in the incidence of 30-day MACE or all-cause mortality when a dual-source CT (without HR control) versus a single-source CT (with HR control) scanner was used for CCTA in acute chest pain patients [15]. No events occurred in either arm during the follow-up period.

Dual-source CCTA was associated with higher image quality and a lower frequency of nondiagnostic examinations but did not have an impact on the rate of downstream revascularization or ICA performed [15]. Furthermore, although the ED length of stay did not differ between groups, dual-source CT had a shorter median CT completion time (95 vs. 117 min, *p* ≤ 0.001).

## 4. Discussion

This review identified several key findings: (1) CCTA had a strong diagnostic accuracy for both the detection of ACS and prediction of MACE; (2) a CCTA-guided pathway was associated with a low risk of future events; and (3) combining CCTA with high-sensitivity cardiac troponin (hs-cTn) testing improved diagnostic performance in patients with inconclusive biomarker levels. To the best of our knowledge, this is the first study to consolidate the evidence from the past decade on the role of CCTA in excluding ACS in low-risk chest pain patients. Since the field of cardiovascular imaging is rapidly evolving, we wanted to explore the recent literature on the use of CCTA in an acute chest pain setting, while taking into consideration the development of more updated technology and diagnostic tools.

CCTA demonstrated an excellent NPV and strong sensitivity for the detection of ACS, which highlights that patients with a negative or normal CCTA can be safely discharged with low risk of future myocardial infarction or unstable angina. This finding is well-aligned with the existing literature. In contrast, PPVs and specificities were generally much lower and less consistent, confirming the relatively low prevalence of ACS that is expected of this patient population. The variable and weak specificity of CCTA may suggest that lesion severity is being overestimated by CCTA. Additionally, further research must be conducted to determine the optimal strategy for managing low-risk chest pain patients with a positive CCTA outcome. Compared to other non-invasive diagnostic strategies, such as stress echocardiography, CCTA demonstrated a stronger diagnostic performance, particularly when a ≥70% stenosis cut-off value was employed. There is limited evidence surrounding the diagnostic accuracy of CCTA with newer generation, dual-source technology, which highlights another area to be explored further.

Beyond anatomical assessment, it is worth highlighting the added value of physiological extensions of CT, such as CT-derived fractional flow reserve (FFR-CT) and CT myocardial perfusion imaging (CT-MPI), in further cardiac investigation when CCTA is positive or inconclusive. Negative FFR-CT is associated with excellent long-term prognosis and low rates of mortality (0%) and MI (0.4%), while a positive FFR-CT predicts obstructive disease requiring revascularization [35]. There is little research to date on the use of CT-MPI in our population of interest; however, given its ability to directly assess ischemia, future studies are likely to highlight its potential in supplementing CCTA-guided pathways in the evaluation of low-risk acute chest pain.

Furthermore, our findings suggest that CCTA can safely discharge patients presenting to the ED with low-risk acute chest pain. When considering only MI, rates of ACS at follow-up were low, ranging from 0% to 3.5% across all studies. When measured as a composite of both MI and UA, the event rate was slightly greater, ranging from 0.5% to 7.8%. Studies that compared CCTA to SOC and stress testing reported that CCTA was equally safe or safer in terms of ACS-related events during the follow-up period. These consistently low event rates reinforce findings from the existing literature, which support the safety of CCTA as a frontline test in the ED.

Understanding the association between CCTA and future MACE was more complex given the heterogeneity in defining MACE, as well as the variability in follow-up lengths across the included studies. Hard events such as MI and cardiac death tended to be infrequent, with revascularizations and readmissions for chest pain typically driving the higher MACE rates recorded in this review. These findings were not unexpected, given that previous studies have noted an association between CCTA and higher rates of revascularization and downstream testing. A meta-analysis conducted by Gongora et al. encompassing 10 RCTs reported that compared to SOC, CCTA had similar rates of MACE, but significantly higher rates of invasive coronary angiography and revascularization [36]. These findings are supported by a living systematic review and meta-analysis of 22 RCTs and nearly 10,000 patients by Barbosa et al., which found no difference in the number of MIs, all-cause deaths, cardiac deaths, or hospitalizations between the CCTA and SOC arms [10]. Although the rate of revascularization was higher in the CCTA arm, costs and length of stay were reduced by 21% and 17%, respectively, which suggests a potential advantage of using CCTA over SOC in this setting. Another study confirmed the ability of CCTA to safely discharge low- to intermediate-risk chest pain patients, with low rates of MACE at 1, 6, and 12 months [37]. CCTA also outperformed exercise stress testing and stress echocardiography in this regard, suggesting that CCTA is a strong alternative to consider in our population of interest. A CCTA-guided strategy could facilitate care in patients with negative or normal outcomes, allowing for more rapid discharge and alleviating burden on emergency departments. Conversely, low-risk acute chest pain patients who are revealed to have positive CCTA results can be promptly referred for further testing, including revascularization or invasive angiography as necessary.

Finally, we sought to understand the future role of CCTA in this patient population in the era of high-sensitivity troponin assays. In a head-to-head comparison of a CCTA-guided versus hs-cTn-guided strategy was conducted, both pathways performed similarly, with no significant differences in future event rates, revascularization, or undetected ACS [28]. This suggests that either strategy can be safely used in this patient population; CCTA was associated with lower direct medical costs and less outpatient testing [DEDIC], which may highlight a possible advantage over hs-cTn assays alone. However, most notably, when used in conjunction with one another (hs-cTn followed by CCTA), the diagnostic accuracy for ACS was significantly improved. These findings indicate that hs-cTn and CCTA may be able to function synergistically to improve clinical decision-making in ambiguous acute chest pain cases—using hs-cTn as an initial screening tool, followed by an anatomical assessment by CCTA for more effective management of low-risk patients.

### Limitations & Clinical Considerations

Some limitations were present in this study. Firstly, while the scoping review methodology provides a summary of the existing literature, it does not assess study quality or aggregate outcomes across studies. Furthermore, there was considerable heterogeneity in the CCTA protocols, populations and defined outcomes of each study. Many studies also highlighted the limited availability of CCTA scanners during off-hours, as well as issues with scan quality (i.e., unreadable or uninterpretable scans/segments), potentially resulting in delayed diagnoses, prolonged emergency department stays, and unnecessary downstream testing. Additionally, it is important to note that many of the studies included in this review were conducted at expert centers, which likely does not reflect the availability of CCTA, patient demographic, or efficiency of patient management in community practice.

Additionally, patients with known CAD, renal dysfunction, atrial fibrillation, prior coronary interventions, or contrast allergies, were systematically excluded. This indicates that CCTA may not be suitable for all groups of patients and should be appropriately targeted towards a specific subset of low-risk patients, until further research has been conducted to evaluate CCTA’s safety in the excluded demographics. However, it is worth highlighting that data included in this review is derived primarily from standard or traditional scanners; newer technology, such as photon-counting CT, is now available and will likely provide a stronger diagnostic performance with fewer limitations. The impact of these advancements on broadening the eligible patient population and improving outcomes will be reflected in future studies.

Another important consideration is the use of CTA in patients with elevated body mass index (BMI). This patient population is typically excluded from studies measuring the efficacy of CTA as diagnostic image quality has been historically difficult to achieve in obese patients (defined as BMI ≥ 30 kg/m^2^) due to high photon attenuation and scatter. Again, despite the emergence of newer generation CT scanners and updated scan protocols, several studies have shown that diagnostic image quality can now be routinely obtained in overweight and obese individuals, suggesting that this is a declining problem [38,39,40,41].

## 5. Conclusions

There is comprehensive evidence to support the role of CCTA in excluding ACS in low-risk acute chest pain patients in the ED setting. The literature highlights the potential of CCTA to facilitate more efficient risk stratification and decision-making in this population due to its excellent negative predictive value and high sensitivity. Thanks to its ability to rapidly and accurately assess and analyze the coronary vessels, CCTA can identify patients who require further care and safely discharge patients with low risk of future ACS and MACE. A combined strategy involving both CCTA and hs-cTn improves the diagnostic accuracy for the detection of ACS and holds promise for the management of low-risk chest pain patients in acute care settings. Further research is needed to determine the effectiveness of CCTA in a broader patient population and to evaluate its integration into a diverse range of multimodal diagnostic pathways.

## Figures and Tables

**Table 1:** Key characteristics of included studies.

Author	Year	Multi-Center	No. Patients	Follow-Up (Median)	Primary CCTA Outcome
Retrospective Studies					
Grunau et al. [14]	2016	Yes	1700	30 days	ACS or MACE at follow-up
Ghoshhajra et al. [13]	2017	No	1022	60 days	ACS or MACE at follow-up
Sturts et al. [17]	2022	No	3816	3 years	ACS or MACE at follow-up
Kim et al. [15]	2023	No	509	30 days	Clinical comparison of dual- vs. single-source CT
Sentagne et al. [16]	2024	No	280	2 months	Diagnostic accuracy of calcifications in diagnosing ACS
Prospective Studies					
Ferencik et al. [20]	2015	Yes (nested in ROMICAT II)	160	-	Diagnostic accuracy of a CCTA and hs-cTn combined strategy in detecting ACS
Mas-Stachurska et al. [22]	2015	No	69	6 months	Diagnostic accuracy of 50% and 70% stenosis in detecting ACS
Mordi et al. [23]	2016	No	232	2 years	ACS or MACE at follow-up
Nabi et al. [24]	2016	No	598	6 months	Diagnostic accuracy of 50% stenosis in predicting MACE
Pena et al. [25]	2016	No (before–after study)	258	30 days	MACE at follow-up
Durand et al. [19]	2017	Yes	217	6 months	Diagnostic accuracy for detection of >50% CAD
Galea et al. [21]	2022	No (pilot study)	104	6 months	ACS or MACE at follow-up
Arslan et al. [18]	2025	Yes	106	30 days	Diagnostic accuracy of ≥50% stenosis to exclude type-1 NSTE-ACS
Randomized Controlled Trials					
Linde et al. [31]	2015	No (CATCH)	600	1 year	MACE at follow-up
Dedic et al. [28]	2016	Yes (BEACON)	500	30 days	ACS or MACE at follow-up
Hollander et al. [29]	2016	Yes (ACRIN)	1392	1 year	ACS or MACE at follow-up
Truong et al. [33]	2016	Yes (ROMICAT II)	1000	28 days	MACE at follow-up
Uretsky et al. [34]	2017	Yes (PERFECT)	411	1 year	ACS or MACE at follow-up
Bamberg et al. [27]	2018	ROMICAT II + ACRIN results	1240	30 days	Diagnostic accuracy for detection of ACS
Levsky et al. [30]	2018	No	400	2 years	ACS or MACE at follow-up
Pineiro-Portela et al. [32]	2021	No	203	4.7 years	ACS or MACE at follow-up
Aziz et al. [26]	2022	No (PROTECCT)	250	12 months	MACE at follow-up

ACS, acute coronary syndrome; CAD, coronary artery disease; CCTA, coronary computed tomography angiography; hs-cTn, high-sensitivity cardiac troponin; MACE, major adverse cardiovascular events; NSTE-ACS, non-ST-elevation acute coronary syndrome.

**Table 2:** Diagnostic accuracy of CCTA.

Author	Year	Follow-Up (Median)	Diagnostic Goal	Sensitivity (%)	Specificity (%)	NPV (%)	PPV (%)	Overall Accuracy (%)
Detection of ACS								
Bamberg et al. [27]	2018	30 days	Detecting ACS in women	94.1	82.7	99.8	18.0	-
			Detecting ACS in men	98.0	84.1	99.8	35.2	-
Mas-Stachurska et al. [22]	2015	6 months	70% stenosis in detecting ACS	100.0	88.4	100.0	73.9	91.3
			50% stenosis in detecting ACS	100.0	76.9	100.0	58.6	82.6
Sentagne et al. [16]	2024	2 months	Calcifications on CCTA in diagnosing ACS	98.4	53.0	99.8	-	-
Prediction of MACE								
Galea et al. [21]	2022	30 days	Absence of obstructive CAD in excluding MACE	-	-	100.0	-	-
Nabi et al. [24]	2016	6 months	50% stenosis in predicting MACE	85	92	100	33	91
Pena et al. [25]	2016	30 days	70% stenosis in predicting MACE	94.7	62.5	83.3	85.7	-
			50% stenosis in predicting MACE	100.0	75.0	100.0	95.8	-
Other Outcomes								
Durand et al. [19]	2017	6 months	Detecting >50% stenosis CAD	96.9	48.3	93.3	67.4	-
Ghoshhajra et al. [13]	2017	60 days	50% stenosis in predicting ICA	-	-	-	79	-

ICA, invasive coronary angiography; NPV, negative predictive value; PPV, positive predictive value. Other abbreviations as in Table 1.

**Table 3:** Acute coronary syndrome at follow-up.

Author	Year	Follow-Up (Median)	CCTA Event Rate (%)	Comparator Event Rate (%)	Comparative Standard	Comments
ACS defined as: Myocardial infarction and/or unstable angina						
Dedic et al. [28]	2016	30 days	0.5 (1/245)	1.2 (3/245)	SOC	
Durand et al. [19]	2017	6 months	0.6 (1/173)	-	-	
Ghoshhajra et al. [13]	2017	60 days	0.5 (5/1022)	-	-	
Linde et al. [31]	2015	1 year	1.8 (5/285)	4.1 (12/291)	SOC (ST or MPI)	All events were UA
Mordi et al. [23]	2016	>6 months	7.8 (18/232)	-	-	
Nabi et al. [24]	2016	6 months	4.6 (13/283)	3.0 (9/300)	SPECT	18/23 ACS events occurred during index admission; group breakdown not reported
Pineiro-Portela et al. [32]	2021	1 year	1.0 (1/100)	0 (0/103)	SE	
Uretsky et al. [34]	2017	1 year	1.4 (3/206)	0.5 (1/205)	ST	
ACS defined as: Myocardial infarction						
Galea et al. [21]	2022	6 months	0.9 (1/104)	-	-	
Grunau et al. [14]	2016	30 days	0 (0/521)	0 (0/1179)	ST	
Hollander et al. [29]	2016	1 year	0.2 (2/883)	0.4 (2/448)	SOC	
Levsky et al. [30]	2018	2 years	3.5 (7/201)	2.0 (4/199)	SE	
Sturts et al. [17]	2022	3 years	0.9 (17/1908)	0.9 (17/1908)	SE	

MPI, myocardial perfusion imaging; SE, stress echocardiography; SOC, standard of care; SPECT, single photon emission computed tomography; ST, stress testing; UA, unstable angina. Other abbreviations as in Table 1.

**Table 4:** Major adverse cardiovascular events at follow-up.

Author	Year	Follow-Up (Median)	MACE Definition	Event Rate (%)	Comments
Arslan et al. [18]	2015	30 days	Death, revascularization	15.1 (16/106)	
Dedic et al. [28]	2016	30 days	Death, ACS, revascularization	10.2 (25/245)	
Durand et al. [19]	2017	6 months	Death, MI, revascularization, readmission for CP	13.8 (24/173)	
Galea et al. [21]	2022	6 months	Cardiac death, nonfatal MI, revascularization, stroke, hospitalization for HF	1.3 (1/76)	
Ghoshhajra et al. [13]	2017	60 days	Cardiac death, MI, revascularization, UA	0.5 (5/1022)	
Grunau et al. [14]	2016	30 days	ACS, PCI, CABG, chest compressions, death	1.3 (7/521)	
Hollander et al. [29]	2016	1 year	Cardiac death, MI	1.4 (12/870)	
Levsky et al. [30]	2018	2 years	Death, MI, stroke, cardiac arrest	5.5 (11/201)	2 deaths occurred, both due to advanced metastatic cancer diagnosed after recruitment
Linde et al. [31]	2015	1 year	Cardiac death, MI, revascularization, readmission for CP, UA	10.5 (30/285)	26/30 events were readmissions for CP
Mas-Stachurska et al. [22]	2015	6 months	Cardiac death, MI, revascularization	4.3 (3/69)	
Mordi et al. [23]	2016	>6 months	Death, non-fatal MI, late revascularization, UA readmission	11.2 (26/232)	
Nabi et al. [24]	2016	6 months	Cardiac death, MI, UA	4.6 (13/283)	18/23 ACS events occurred during index admission; group breakdown not reported
Pena et al. [25]	2016	30 days	MI, revascularization, cardiac death	0% (0/128)	
Pineiro-Portela et al. [32]	2021	4.7 years	Death, non-fatal MI, revascularization, readmission	29.0 (29/100)	
Sturts et al. [17]	2022	3 years	MI, revascularization	0.9 (MI); 2.7 (revascularization)	No composite MACE rate reported
Truong et al. [33]	2016	28 days	Death, MI, UA, revascularization	0.4 (2/501)	
Uretsky et al. [34]	2017	1 year	Cardiac death, all-cause mortality, MI, UA	1.4 (3/206)	

CABG, coronary artery bypass graft; CP, chest pain; HF, heart failure; MI, myocardial infarction; PCI, percutaneous coronary intervention; UA, unstable angina. Other abbreviations as in Table 1.

## Data Availability

No new data were created or analyzed in this study. Data sharing is not applicable to this article.

## Supplementary Materials

The following supporting information can be downloaded at: https://www.mdpi.com/article/10.3390/jcdd12120493/s1, Table S1: CT protocols of included studies; Table S2: Patient characteristics in CCTA studies; Table S3: Patient characteristics in comparative studies (CCTA vs. other testing modalities).

## Abbreviations

The following abbreviations are used in this manuscript:

ACS	Acute coronary syndrome
CAD	Coronary artery disease
CCTA	Coronary computed tomography angiography
ECG	Electrocardiogram
ED	Emergency department
Hs-cTn	High-sensitivity cardiac troponin
MACE	Major adverse cardiovascular events
NPV	Negative predictive value
PPV	Positive predictive value
SE	Stress echocardiography
SOC	Standard of care
