# Supplementary material for: Coronary Computed Tomography Angiography to Exclude Acute Coronary Syndrome in Low-Risk Chest Pain Patients
Source: J Cardiovasc Dev Dis. 2025 Dec 14;12(12):493. doi: 10.3390/jcdd12120493 (PMC12733454; doi:10.3390/jcdd12120493)
Supplement: Supplementary file 1 [file jcdd-12-00493-s001.zip › jcdd-3947715-supplementary.pdf]

Table S1. CT protocols of incuded studies.

Study	Year	CT Technology	Acquisition Method	Tube Settings (voltage, current)
Arslan et al. [18]	2025	Single-source and dual-source *(Aquilion One Prism Edition 320-Detector, Canon Medical Systems; Somatom Force/Somatom Definition Flash, Siemens Medical Solutions)*	Prospective ECG-gated	Automatic kV
Aziz et al. [26]	2022	Dual-source *(Siemens Medical Solutions)*	ECG-synchronized	-
Bamberg et al. [27]	2018	≥64-slice	-	-
Dedic et al. [28]	2016	≥64-slice	ECG-synchronized	-
Durand et al. [19]	2017	64-slice	ECG-synchronized	-
Ferencik et al. [20]	2015	≥64-detector	Retrospective or prospective ECG-gated	-
Galea et al. [21]	2022	Single-source, 320-detector *(Aquilion One Vision Edition, Canon Medical Systems)*	-	100–120 kV, automatic current modulation
Ghoshhajra et al. [13]	2017	Dual-source, 256-slice *(Somatom Definition Flash or Somatom Force; Siemens Medical Solutions)*	Retrospective or prospective ECG-gated	120 kV
Grunau et al. [14]	2016	-	-	-
Hollander et al. [29]	2016	≥64-slice	ECG-gated	-
Kim et al. [15]	2023	Single-source, 64-detector; dual-source, 96-detector *(Revolution EVO CT, General Electric; Somatom Force, Siemes Medical Solutions)*	Retrospective or prospective ECG-gated	SSCT: 80–120 kV, 398–600 mA<br>DSCT: 70–120 kV, 131–601 mA
Levsky et al. [30]	2018	Single-source, 64-detector	Performed according to established guidelines. (Abbara S, Blanke P, Maroules CD, et al. SCCT guidelines for the performance and acquisition of coronary computed tomographic angiography: a report of the Society of Cardiovascular Computed Tomography Guidelines Committee: endorsed by the North American Society for Cardiovascular Imaging (NASCI). J Cardiovasc Comput Tomogr 2016;10:435–49.)	-
Linde et al. [31]	2015	Single-source, 320-detector *(Aquilion One, Toshiba America Medical Systems)*	Performed according to established guidelines. (Leipsic J, Abbara S, Achenbach S, et al. SCCT guidelines for the interpretation and reporting of coronary CT angiography: a report of the Society of Cardiovascular Computed Tomography Guidelines	-

			Committee. J Cardiovasc Comput Tomogr 2014;8:342–58.)	
Mas-Stachurska et al. [22]	2015	Single-source, 64-slice *(Sensation 64, Siemens Medical Solutions)*	Retrospective ECG-gated	120 kV, 550–850 mA
Mordi et al. [23]	2016	Dual-source, 64-slice *(Discovery 750 HD, General Electric)*	Retrospective or prospective ECG-gated	100–140 kV, 370–412 mA
Nabi et al. [24]	2016	64-slice *(Phillips)*	Retrospective or prospective ECG-gated	-
Pena et al. [25]	2016	Single-source, 64-detector *(LightSpeed VCT, General Electric)*	Retrospective ECG-gated	120 kV, 400–800 mA
Pineiro-Portela et al. [32]	2021	64-detector	30%-70% of RR interval	-
Sentagne et al. [16]	2024	-	-	-
Sturts et al. [17]	2022	-	-	-
Truong et al. [33]	2016	≥64-slice	Retrospective or prospective ECG-gated	-
Uretsky et al. [34]	2017	Single-source, 64-detector *(Aquilion 64, Toshiba America Medical Systems; Somatoform Sensation 64, Siemens Medical Solutions)*	Contrast enhanced ECG-gated	-

Table S2. Patient characteristics in CCTA studies.

Study	Year	No. patients	Inclusion Criteria	Exclusion Criteria	Age (mean±SD)	Gender (%F)	HT (%)	Dyslipidemia (%)	DM (%)	FHx (%)	Smoker (%)
Arslan et al. [18]	2025	106	- Age 30-80 yrs<br>- Acute CP suggestive of NSTE-ACS<br>- Inconclusively elevated hs-cTn	- History of CAD<br>- ICA/CCTA within past 3 years<br>- Clinical instability<br>- CCTA contraindications (contrast allergy, renal insufficiency, pregnancy, etc.)	65 ± 10	29 (31/106)	55 (58/106)	47 (50/106)	26 (27/106)	35 (37/106)	58 (61/106)
Bamberg et al. [27]	2018	1240	- Age 30-74 yrs<br>- Acute CP suggestive of ischemia<br>- Low-intermediate risk (negative ECG, troponin)	- Clinical instability<br>- CCTA contraindications	51 ± 8.9	49 (612/1240)	52 (645/1240)	34 (426/1240)	15 (183/1240)	28 (353/1240)	50 (611/1240)

Durand et al. [19]	2017	217	- Age >18 yrs<br>- CP with non-diagnostic ECG, two normal troponin measurements	NR	57 ± 11.2	34 (73/217)	44 (96/217)	54 (118/217)	10 (21/217)	40 (86/217)	30* (66/217)
Ferencik et al. [20]	2015	160	- Patients in the ROMICAT II trial in the CCTA arm<br>- CP suggestive ACS with negative ECG and conventional troponins	- Patients without hsTn-I measurements	53 ± 7.6	40 (64/160)	52 (83/160)	44 (71/160)	13 (21/160)	31 (50/160)	53 (85/160)
Galea et al. [21]	2022	104	- Age >18 yrs<br>- Acute CP of possible cardiac origin<br>- Low risk (HEART score 0-3)	- Known CAD<br>- Prior revascularization<br>- CCTA contraindications<br>- Extensive coronary calcifications	63 ± 11.2	35 (36/104)	52 (54/104)	48 (50/104)	10 (10/104)	32 (33/104)	45 (47/104)
Ghoshhajra et al. [13]	2017	1022	- CCTA on ED visit during study period<br>- Negative ECG and troponin levels	- CCTA contraindications	53 ± 11.0	42 (433/1022)	40 (410/1022)	28 (288/1022)	12 (118/1022)	18 (181/1022)	22* (223/1022)
Hollander et al. [29]	2016	1368	- Age >30 years<br>- Acute CP suggestive of ACS<br>- Non-diagnostic ECG<br>- TIMI 0-2	- CCTA contraindications	47	53 (723/1368)	51 (694/1368)	27 (367/1368)	14 (194/1368)	29 (394/1368)	33* (446/1368)
Mas-Stachurska et al. [22]	2015	69	- Presentation to ED with angina<br>- Negative ECG and troponin levels<br>- Additional inclusion criteria: Age >35 yrs with pretest probability of CAD >10%	- CCTA contraindications	61 ± 11.2	36 (25/69)	67 (46/69)	58 (40/69)	9 (6/69)	29 (20/69)	51 (35/69)
Mordi et al. [23]	2016	232	- Acute CP<br>- Non-diagnostic ECG and negative troponin<br>- Intermediate pretest probabiliity of CAD (15-85%)	- CCTA contraindications	54 ± 10.9	58 (134/232)	41 (96/232)	53 (124/232)	7 (17/232)	22 (52/232)	13* (31/232)

Sentagne et al. [16]	2024	280	- Typical CP or atypical CP of suspected cardiac origin<br>- Non-diagnostic ECG and negative troponin	NR	61.5	46 (130/280)	47 (132/280)	40 (113/280)	24 (67/280)	9 (24/280)	42* (119/180)

CP, chest pain; ECG, electrocardiography; NSTE-ACS, non-ST-elevation acute coronary syndrome; TIMI, thrombolysis in myocardial infarction score. Other abbreviations as in Table 1. *Only includes current smokers.

Table S3. Patient characteristics in comparative studies (CCTA vs other testing modalities).

Study	Year	No. patients	Inclusion Criteria	Exclusion Criteria	Age (mean±SD)	Gender (%F)	HT (%)	Dyslipidemia (%)	DM (%)	FHx (%)	Smoker (%)
Aziz et al. [26]	2022	CCTA =125	- CP suggestive of ACS<br>- Non-diagnostic ECG and troponin	- AFib<br>- Haemodynamic instability<br>- History of CAD, coronary anomalies, congenital heart disease<br>- Prior coronary revascularization<br>- CCTA contraindications (contrast allergy, renal insufficiency, pregnancy, etc.)	55 ± 13.3	26 (32/125)	45 (56/125)	42 (52/125)	19 (24/125)	28 (35/125)	47 (59/125)
		SOC = 125			56 ± 14.0	23 (30/125)	47 (59/125)	40 (50/125)	18 (23/125)	26 (32/125)	50 (62/125)
Dedic et al. [28]	2016	CCTA = 250	- Age 30-75 yrs for men, 30-80 yrs for women<br>- Acute CP suggestive of ACS	- History of CAD<br>- BMI >40kg/m^2<br>- CCTA contraindications	55 ± 10	49 (123/250)	-	-	12 (31/250)	45 (112/250)	47 (118/250)
		SOC = 250			53 ± 9	45 (113/250)	-	-	13 (33/250)	39 (98/250)	40 (100/250)
Gruna u et al. [14]	2016	CCTA = 521	- Age 18-65 yrs<br>- Acute CP with cardiac features	- Elevated biomarkers and/or ischemic ECG changes<br>- History of acute reactive airway disease<br>- CCTA contraindications	51	38 (199/521)	36 (186/521)	21 (110/521)	10 (53/521)	18 (93/521)	30* (154/521)
		EST = 1179			51	44 (524/1179)	33 (378/1179)	20 (231/1179)	10 (110/1179)	21 (150/1179)	29* (315/1179)
Kim et al. [15]	2023	SSCT = 205	- Age >19 yrs<br>- Low-intermediate risk of acute CP in ED based on HEART score	- Prior coronary revascularization	52	42 (87/205)	22 (45/205)	10 (21/205)	10 (20/205)	5 (10/205)	12* (25/205)
		DSCT = 405			52	46 (139/304)	27 (83/304)	8 (24/304)	8 (25/304)	2 (7/304)	8* (25/304)

Levsky et al. [30]	2018	CCTA = 201	- Low-intermidiate acute CP in ED	- CCTA contraindications<br>- SE contraindications<br>- Known CAD	55 ± 9.2	43 (87/201)	54 (109/201)	45 (91/201)	29 (58/201)	35 (70/201)	25* (51/201)
		SE = 199			54 ± 10	42 (83/199)	60 (119/199)	43 (85/199)	28 (55/199)	35 (69/199)	24* (48/199)
Linde et al. [31]	2015	CCTA = 285	- Age >18 yrs<br>- Low-intermediate pre-test probability of CAD<br>- Normal ECG and 2 normal troponin tests	- CCTA contraindications	56 ± 12	44 (124/285)	47 (135/285)	41 (117/285)	12 (35/285)	24 (69/285)	60 (172/285)
		SOC = 291			55 ± 12	42 (123/291)	36 (106/291)	35 (101/291)	10 (29/291)	26 (76/291)	67 (195/291)
Nabi et al. [24]	2016	CCTA = 288	- Age >18 yrs- Acute CP without any initial troponin elevation	- History of CAD- Known cardiomyopathy- CCTA contraindications	54 ± 12.5	55 (158/288)	50 (144/288)	39 (113/288)	15 (42/288)	25 (71/288)	26* (77/288)
		SPECT = 310			53 ± 11.9	56 (174/310)	51 (157/310)	37 (115/310)	15 (48/310)	21 (66/310)	27* (85/310)
Pena et al. [25]	2016	CCTA = 128	- Age >25 yrs<br>- Low-intermediate risk acute CP<br>- Normal ECG and troponin	- Hypotension<br>- Known CAD<br>- CCTA contraindications	57 ± 11.7	37 (47/128)	22 (26/128)	26 (31/128)	3 (4/128)	31 (37/128)	32 (39/128)
		SOC = 130			57 ± 14.3	38 (50/130)	39 (51/130)	29 (37/130)	9 (12/130)	18 (24/130)	30 (38/130)
Pineiro-Portela et al. [32]	2021	CCTA = 100	- Age 18-80 yrs<br>- ≥1 cardiovascular risk factor<br>- Suspicion of ACS<br>- Non-diagnostic ECG and troponin	- History of stent implantation<br>- CCTA contraindications	64 ± 11	35 (35/100)	71 (71/100)	74 (74/100)	27 (27/100)	6 (6/100)	39 (39/100)
		SE = 103			64 ± 11	36 (37/103)	70 (72/103)	76 (78/103)	29 (30/103)	4 (4/103)	34 (35/103)
Sturts et al. [17]	2022	CCTA = 1908	- Acute CP in the ED during defined study period<br>- Underwent CCTA/SE within 72 hrs of initial encounter	NR	47	52 (994/1908)	48 (918/1908)	44 (831/1908)	16 (315/1908)	-	-
		SE = 1908			49	55 (1042/1908)	50 (944/1908)	42 (798/1908)	16 (309/1908)	-	-

Truong et al. [33]	2016	Diabetes = 173	- Age 40-74 yrs<br>- Acute CP without ischemic ECG changes or initial positive troponin	- Known CAD<br>- BMI >40 kg/m^2<br>- CCTA contraindications	57 ± 8.2	55 (96/173)	88 (152/173)	74 (128/173)	-	21 (26/173)	46 (79/173)
		No diabetes = 827			54 ± 8.0	45 (372/827)	47 (389/827)	39 (326/827)	-	28 (235/827)	50 (413/827)
Uretsky et al. [34]	2017	CCTA = 206	- Age ≥45 yrs<br>- CP in the ED<br>- Negative initial troponin I and ECG	- Known CAD<br>- CCTA contraindications	59 ± 10	54 (111/206)	68 (140/206)	43 (88/206)	24 (50/206)	18 (37/206)	45* (92/206)
		ST = 205			60 ± 10	53 (108/205)	69 (142/205)	53 (109/205)	33 (68/205)	25 (51/205)	46* (93/205)

BMI, body mass index; CP, chest pain; ECG, electrocardiography; NSTE-ACS, non-ST-elevation acute coronary syndrome; TIMI, thrombolysis in myocardial infarction score. Other abbreviations as in Table 1. *Only includes current smokers.
